# Supplementary material for: Predicting visual function by interpreting a neuronal wiring diagram
Source: Nature. 2024 Oct 2;634(8032):113–23. doi: 10.1038/s41586-024-07953-5 (PMC11446822; doi:10.1038/s41586-024-07953-5)
Supplement: Supplementary file 6 — CRF and ERF predictions for individual TmY4 and TmY9 cells. Analogous to Supplementary Data 3, but for TmY target types. Shown are the top four monosynaptic pathways, the strongest pathway passing through each of the top ten intermediary types (ranking from Extended Data Fig. 7), and the trisynaptic pathway Tm1–TmY–Dm3–TmY (see the section entitled Prediction of spatial normalization). [file 41586_2024_7953_MOESM6_ESM.zip › DataS4/TmY9q/720575940620680408.pdf]

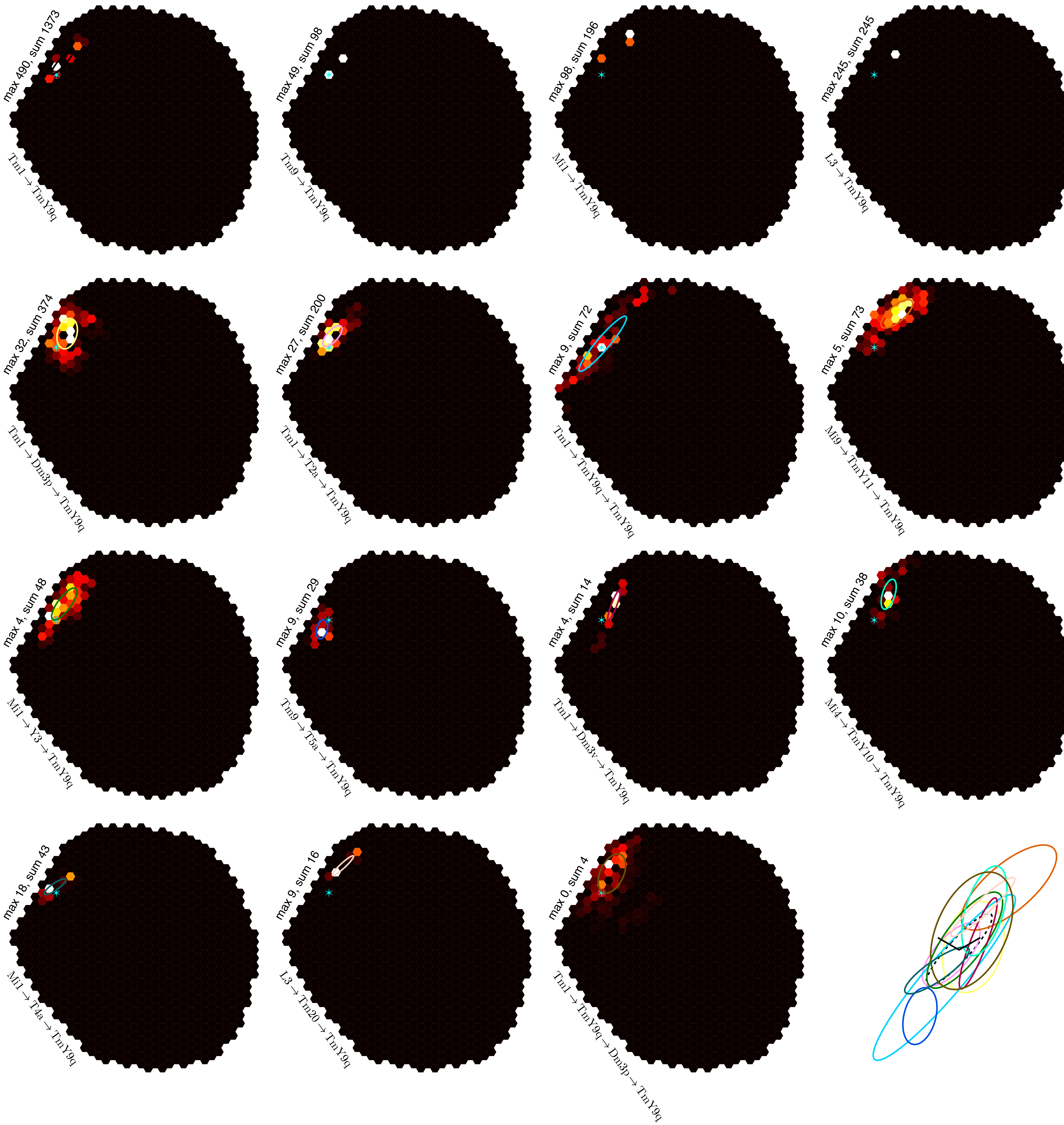

max 490, sum 1373
Tm1 → TmY9q
max 49, sum 98
Tm9 → TmY9q
max 98, sum 196
Mi1 → TmY9q
max 245, sum 245
L3 → TmY9q
max 32, sum 374
Tm1 → Dm3p → TmY9q
max 27, sum 200
Tm1 → T2a → TmY9q
max 9, sum 72
Tm1 → TmY9q → TmY9q
max 5, sum 73
Mi9 → TmY11 → TmY9q
max 4, sum 48
Mi1 → Y3 → TmY9q
max 9, sum 29
Tm9 → T5a → TmY9q
max 4, sum 14
Tm1 → Dm3v → TmY9q
max 10, sum 38
Mi4 → TmY10 → TmY9q
max 18, sum 43
Mi1 → T4a → TmY9q
max 9, sum 16
L3 → Tm20 → TmY9q
max 0, sum 4
Tm1 → TmY9q → Dm3p → TmY9q
